# An Evaluation of the Structure of an Integrated Regional Remote Care Management Program for Patients with Selected Chronic Diseases in Canada

**DOI:** 10.5334/ijic.8629

**Published:** 2026-02-05

**Authors:** Diedron Lewis, Karin Swift, Sarah Weberman

**Affiliations:** 1 Dalla Lana School of Public Health, University of Toronto, Ontario, Canada; 2 Mississaugua Health Ontario Health Team, Mississaugua, Ontario, Canada; 3 Connected Care Halton Ontario Health Team, Halton, Ontario, Canada

**Keywords:** remote care management, program evaluation, digital health

## Abstract

**Background:**

Since the onset of the COVID-19 pandemic, the number of Remote Care Management (RCM) programs in Ontario has increased; however, many have not been objectively evaluated based on their structure and performance.

**Objective:**

This study demonstrates how the core structure of the RCM model, developed by the Connected Care Halton Ontario Health Team (CCHOHT) with Halton Healthcare Services and Ontario Health atHome, aligns with the RCM recommendations and evaluation tool from the Ontario Ministry of Health (OMH). The focus is specifically on the CCHOHT’s RCM program for chronic diseases.

**Method:**

This study presents the CCHOHT’s RCM model to demonstrate the degree to which it is consistent with the recommendations for developing RCM programs forwarded by the OMH. It also evaluates the CCHOHT’s current chronic diseases RCM program using the OMH’s RCM evaluation taxonomy tool and an amended version of this tool proposed by the authors. The taxonomy is based on four foundational criteria: technology, touch, integration and equity.

**Results:**

The CCHOHT’s chronic diseases RCM program meets the OMH’s four RCM taxonomy criteria. The program is a digital solution that remotely manages patients, provides follow-up communication, and escalates critical cases through a more patient-centred, connected clinical pathway of services and care partners.

**Conclusion:**

The CCHOHT’s RCM model and its chronic diseases RCM program are built on intersecting principles of RCM and integrated care as articulated by the OMH. The adaptable nature of the RCM model allows it to be extended to other clinical conditions.

## Introduction

Remote Care Management (RCM) is a telehealth-based approach to delivering healthcare to patients that uses telecommunication technologies to monitor and assess patient conditions, and escalate care, when necessary [1,2,3]. RCM programs may target patients with both chronic and acute conditions, including those with respiratory diseases, diabetes, palliative cases, and those in surgical transition [4,5]. New dispensations of such programs now accommodate features for more comprehensive and personalized disease management through health surveillance, remote communication between patient and care team, data collection, and greater patient autonomy in managing health conditions [4,5]. Digital devices typically include tools to measure, monitor and report weight, blood pressure, blood glucose, and blood oxygen levels, as well as medication dispensing systems to support medicine adherence [5,6]. With anticipated benefits including improved patient care experiences and health outcomes, fewer emergency department (ED) visits and hospital admissions, and lower healthcare costs and expenses for patients, it is expected that remote management will become an integral part of modern health systems [4,5,7].

The expansion of remote care management in Ontario, and Canada as a whole, has been motivated in part by the challenges posed by the COVID-19 pandemic, which called for innovative solutions to delivering healthcare in an environment of limited social and physical contact [4]. This resulted in the establishment of several RCM programs across the province that are responsible for identifying and managing individuals in need of medical intervention at a much earlier stage to limit the number of more critical cases presenting at hospitals and other care institutions [2,4].

This situation is also true for Halton Region, Ontario, Canada, where the Connected Care Halton Ontario Health Team (CCHOHT) in partnership with Halton Healthcare Services and Ontario Health atHome (formally known as Home and Community Care Support Services), developed an RCM program in February 2022 to virtually manage patients diagnosed with chronic conditions, namely COVID-19, Chronic Obstructive Pulmonary Disease (COPD), Congestive Heart Failure (CHF), pneumonia, Interstitial Lung Disease (ILD), asthma, and pulmonary embolism (PE). Hospital data within the region also point to the need to establish such an intervention. Firstly, acute care discharges associated with these conditions increased by 33.4% from 1,840 patients to 2,455 patients between 2019/20 and 2021/22 [8]. Secondly, the average length of stay for patients during this period was 9.4 days, while the expected length of stay was 7.3 days [8]. The RCM program is, therefore, envisioned as a digitally based intervention to facilitate the transition of patients to home care while providing them with the tools to better manage their conditions remotely and limit the number of avoidable visits to the ED and hospital admissions.

The CCHOHT’s RCM program, however, has not been previously evaluated against evidence-based principles and best practices. This type of analysis is valuable for assessing whether the RCM program is rooted in integrated care and well-positioned to benefit from the rewards of an integrated approach. The chronic diseases RCM program is highlighted in this analysis because it is the most established of the RCM programs at CCHOHT, and it provides an opportunity for program organizers to meaningfully reflect on its establishment and overall performance.

The objective of this study is to compare the core structure of the RCM model and, by extension, the chronic diseases RCM program developed by the CCHOHT according to best practices proposed by the Ontario Ministry of Health (OMH), as reflected in the OMH’s RCM recommendations, its RCM taxonomy evaluation matrix, and an amended version of this matrix proposed by the authors. The taxonomy is a tool that enables decision makers to identify components of an RCM program that meet best practice standards and address those that may require additional investment and reimagining [4].

## Method

This study compared the RCM model established by the CCHOHT to best practice principles for building RCM programs outlined by the OMH. These principles state that RCM programs should:

1. *Support adaptive approaches in the implementation and sustainment of RCM programs*.
2. *Adopt a patient-centric and individualized approach that includes customizable RCM features based on patient needs*.
3. *Program length of stay should be modifiable, as many patients with complex health needs require longer-term care*.
4. *Include patients and caregivers in the development of RCM programs through co-design or participatory design methods*.
5. *Integrate RCM programs with existing services and resources within and across Ontario Health Teams*.
6. *Embed RCM programs seamlessly into existing workflows to promote staff buy-in and improve staff retention*.
7. *Develop a streamlined referral and onboarding process with partners and referral sites*.
8. *Remove barriers and reduce health inequities to improve the accessibility of RCM programs and, more generally, health care services* [4].

The structure of the CCHOHT’s chronic diseases RCM program was then reviewed based on the RCM taxonomy and rubric developed by the OMH. The taxonomy provides a basis for evaluating the effectiveness of an RCM program in terms of its impact on the healthcare system and population health [4]. It is used as a tool to pragmatically assess the performance of RCM programs, even though such programs may serve different population needs and have varying characteristics in terms of their digital component, clinical flow, medical interventions, staffing, and resource needs [4]. The taxonomy also provides a systematic approach to designing, implementing, and administering RCM programs [4].

The RCM taxonomy comprises 12 characteristics, grouped into four domains: A = Technology, B = Touch, C = Integration, and D = Equity/Patient-Centricity. Technology refers to the level of automation and technical complexity of the RCM platform. Touch speaks to the level of monitoring and interaction required between the patient and the RCM team. Integration captures the extent to which the RCM program is linked to (or leverages) existing systems, including services, resources, workflows, and infrastructure. Meanwhile, equity/patient-centricity accounts for the extent to which the RCM program proactively enables inclusion, equitable access, and/or patient-centricity [4]. Table S1 in the supplementary file provides a detailed description of these characteristics and domains.

For the evaluation, Domains A (Technology) and B (Touch) are grouped, while Domains C (Integration) and D (Equity/Patient-Centricity) are also grouped [4]. Each domain is characterized based on the available program features and is rated as either low or high [4]. This results in 16 individual classifications from which an existing RCM program can be described, ranging from 1A, which represents an ideal RCM program, to 4D, which is the least ideal [4]. Table S2 in the supplementary file presents the grouping of the domains, the scoring options, and the RCM typologies.

The process of rating the characteristics of each domain is based on the rubric presented in Table S3 in the supplementary file. Each program characteristic is assigned a score between 0 and 3 and is also given a rating from high to low, depending on how and whether an RCM program has adopted a particular feature.

### Amended RCM taxonomy evaluation tool

The authors also revised the OMH’s rubric to address methodological concerns and facilitate a more balanced approach to the evaluation process. The original OMH rubric presented limitations in scoring balance and descriptor clarity. Specifically, several characteristics lacked defined descriptors for moderate scores (e.g., domains A1, A3, B2, and C2), and intervals to classify a program as high, moderate, or low are not defined. These concerns pose a challenge in calculating aggregate and average scores and in classifying programs with nuanced features. Additionally, the taxonomy matrix did not account for moderate classifications, despite their partial presence in the rubric.

The authors propose addressing these challenges by amending the initial rubric to ensure it has a more balanced structure, where each program feature is defined and scaled according to its operational relevance.

This revision was done intuitively, as described by Fulcher (2003) and Hawkey and Barker (2004) [9,10], relying on the rubric that was previously developed and the literature on digital health evaluation frameworks [11], along with consultations with clinicians and program coordinators.

The updated rubric is presented in Table 1, where some descriptors were re-categorized. Specifically, “Manual” under A1 (Alert Protocol) was reclassified from low to moderate to reflect its functional adequacy in certain contexts. “Fragmented” under A3 (Data Access) was shifted from low to moderate to acknowledge partial integration. “Moderately to highly specialized” was introduced under B2 (Monitoring Specialization) to better reflect team composition. “Multiple separate” under C2 (Device Linkages) was reclassified from low to moderate to recognize partial interoperability. The authors believe that these adjustments in the categories were sufficiently reasonable.

**Table 1:** Updated Rubric for evaluating RCM domains and characteristics.


DOMAINS/CHARACTERISTICS	HIGH (3)	MODERATE (2)	LOW (1/0)

Domain A: Technology			

A1. Alert protocol	[Automatic]	[Manual]	[None] or [Unknown]

A2. Data entry modality	[Fully automated]	[Semi-automated]	[Manual] or [Unknown] or [None]

A3. Data access	[Centralized]	[Fragmented]	[None] or [Unknown]

A4. Manual data entry (frequency)	[Monthly]	[Weekly] or [Bi-weekly]	[Daily] or [Unknown] or [None]

Domain B: Touch			

B1. Follow-up communication	[Synchronous on demand]	[Asynchronous on demand]	[Pre-scheduled] or [None] or [Unknown]

B2. Level of monitoring specialization	[Moderately to highly specialized]	[Low specialization]	[No specialization] or [None] or [Not applicable]

B3. Availability of team	[24/7] or [Regular + weekends]	[Regular workdays]	[Irregular] or [None] or [Not applicable] or [Unknown

B4. Risk profile	[High]	[Moderate]	[Low] or [Non-specific] or [Unknown]

Domain C: Integration			

C1. Integration consideration Services and resources Workflows Systems and infrastructure	[All three features]	[Any two features]	[0–1 feature] or [Unknown]

C2. Device linkages	[Multiple linked] or [Single]	[Multiple separate]	[None] or [Unknown]

Domain D: Equity/Patient-Centricity			

D1. Device ownership	[System provided]	[Mixed ownership]	[BYOD] or [None] or [Unknown]

D2. Equity consideration Language inclusivity Digital literacy Offline functionality Cultural adaptability Digital access Access to health information	[5–6 features]	[3–4 features]	[1–2 features] or [None] or [Unknown]


Source: Centre for Digital Health Evaluation, Women’s College Hospital Institute for Health System Solutions and Virtual Care (2023) “Remote Patient Monitoring: Evaluation Final Report” and authors’ input.

This updated rubric is also amenable to the new taxonomy matrix being proposed, which now fully accounts for a moderate option that describes associated elements of an RCM program (see Table 2). The matrix is expanded and now contains 89 options for which an RCM program can be categorized. While this may seem superfluous, the matrix should be viewed with the following caveats, which make it more palatable.

**Table 2:** Updated RCM taxonomy matrix according to type and group.


			GROUP																	

		GROUP A			GROUP B		GROUP C		GROUP D		GROUP E		GROUP F		GROUP G		GROUP H		GROUP I	
								
			TECH	TOUCH	TECH	TOUCH	TECH	TOUCH	TECH	TOUCH	TECH	TOUCH	TECH	TOUCH	TECH	TOUCH	TECH	TOUCH	TECH	TOUCH

			High	High		High	High				High			High						

					Mod			Mod	Mod	Mod						Mod	Mod			

												Low	Low		Low			Low	Low	Low

TYPE	Type 1																			

	Integration	Equity	1A		1B		1C		1D		1E		1F		1G		1H		1I	

	High	High																		

	

	

	Type 2																			

	Integration	Equity	2A		2B		2C		2D		2E		2F		2G		2H		2I	

	High																			

		Mod																		

	

	Type 3																			

	Integration	Equity	3A		3B		3C		3D		3E		3F		3G		3H		3I	

		High																		

	Mod																			

	

	Type 4																			

	Integration	Equity	4A		4B		4C		4D		4E		4F		4G		4H		4I	

	

	Mod	Mod																		

	

	Type 5																			

	Integration	Equity	5A		5B		5C		5D		5E		5F		5G		5H		5I	

	High																			

	

		Low																		

	Type 6																			

	Integration	Equity	6A		6B		6C		6D		6E		6F		6G		6H		6I	

		High																		

	

	Low																			

	Type 7																			

	Integration	Equity	7A		7B		7C		7D		7E		7F		7G		7H		7I	

	

	Mod																			

		Low																		

	Type 8																			

	Integration	Equity	8A		8B		8C		8D		8E		8F		8G		8H		8I	

	

		Mod																		

	Low																			

	Type 9																			

	Integration	Equity	9A		9B		9C		9D		9E		9F		9G		9H		9I	

	

	

	Low	Low																		


Source: Centre for Digital Health Evaluation, Women’s College Hospital Institute for Health System Solutions and Virtual Care (2023) “Remote Patient Monitoring: Evaluation Final Report” and authors’ input.

1. The combination High-High is the most ideal RCM program feature.
2. The combinations High-Medium and Medium-High are indistinguishable in terms of their ranking.
3. The combinations High-Low and Low-High are indistinguishable in terms of their ranking.
4. The combinations Medium-Low and Low-Medium are indistinguishable in terms of their ranking.
5. The combination Low-Low is the least favourable RCM program feature.

We also provided the following scoring intervals that define the limits of each descriptive category:

Low: 0–1.499

Moderate: 1.5–2.499

High: 2.5–3.0

The updated rubric and taxonomy matrix were reviewed and endorsed by two representatives of the Patient Family and Caregiver group affiliated with the CCHOHT. They also independently validated the scores assigned during the evaluation.

## Description of care of practice

### CCHOHT RCM model

The remote management model developed by CCHOHT was borne out of the need to administer care in an environment of limited physical contact brought on by the COVID-19 pandemic. It is intended to facilitate the transition of patients to home care and reduce the number of avoidable hospital encounters. It aligns with the Ontario Public Health Standards, which encourages public health organizations across Ontario to identify priority populations in need of public health intervention by assessing population health needs in various jurisdictions across the province [8]. Priority populations are defined as population groups that are more likely to experience an increased disease burden or unfavourable health outcomes or face challenges in accessing appropriate care and would benefit from targeted public health interventions [9,10].

In addressing the aforementioned issues, CCHOHT developed an RCM model that proposes a digital-based intervention in the delivery of healthcare to high-priority ambulatory patients. The model utilizes digital technologies to provide remote care to patients, whether it be to assist in the recovery from illness and/or manage disease and symptoms among targeted patient populations. Patients are provided with appropriate devices to measure and record key physiological indicators, including weight, oxygen, blood pressure, and blood glucose levels. Patient records are monitored and assessed by a member of the patient’s medical care team. This person also provides counsel and care support when necessary to the patient and escalates more serious cases to higher levels of care to limit the number of non-urgent cases presenting at emergency departments.

The model also recognizes the value of leveraging existing care pathways and resources. For CCHOHT, this means assessing points along the flow of care where patients can benefit from remote management. This requires establishing strategic connections with existing care providers to promote patient referrals into specific RCM programs and patient transfers to other levels of care, when needed.

A key component of the RCM model is increased patient autonomy in care management. Patients enrolled in an RCM program are provided with the requisite resources (remote access to a care team, education material) and training to better manage their condition at home. Patients, along with other key care partners (family/caregivers), are also involved in the co-design of RCM programs.

Additionally, the model’s uniqueness lies in its ease of adaptation to a range of clinical pathways for ambulatory patients. At present, CCHOHT has developed an RCM program based on its RCM model, targeting patients with chronic diseases and those in need of early palliative care.

### CCHOHT chronic diseases RCM program

We first describe the chronic diseases RCM program. This is then used to justify the scores awarded to each RCM program feature.

The chronic diseases RCM program is a digital care program based on CCHOHT’s RCM model that targets patients with specific chronic conditions who require assistance with managing their health at home in Halton Region, Ontario. All patients with at least one of the following diagnoses who are referred to home care and can check their vital signs independently or with the help of a caregiver are eligible to enrol in the program: COVID-19, CHF, COPD, ILD, pulmonary embolism, pneumonia, and asthma.

The program is embedded into the clinical pathways of each respective ailment listed above. It relies on strategic alliances with key partners in these pathways to function, namely CCHOHT, Ontario Health atHome (that provides home care services), Halton Healthcare Services (the local hospital system), the patient’s family doctor and/or cardiologist, a local network of doctors, community paramedic services, the patient, and the patient’s family and caregiver.

Patients who are referred to the program and who meet the criteria for home care (as mentioned earlier) are encouraged to participate in the RCM program by their assigned care team. Patient referrals to home care are accepted from various care providers, including outpatient clinics, family doctors, hospitals, and home care services, as well as patients, family/caregivers, and community organizations within Halton Region.

A patient’s care team typically consists of a physician, a rapid response nurse (RRN), and the RCM monitoring team, with additional support services from care providers within Halton Region, along with the patient, family/and caregivers. Patients have the option to join the program with their family physician or have one assigned to them from a local network of doctors. Patients are also assigned an RRN from Ontario Health atHome who provides care support throughout the home care journey. This includes onboarding the patient onto the RCM program by setting up all devices and training the patient to use them. Meanwhile, patient monitoring is conducted by the RCM monitoring team, which comprises program coordinators, clinical coordinators, nurses, paramedics, and respiratory therapists.

Patients are provided with a blood pressure cuff, an oximeter, a scale, and a tablet as part of their onboarding package onto the RCM program. They are encouraged to use these devices to check and record three physiological indicators daily, i.e. blood pressure level, blood oxygen level, and weight (for patients with CHF). The tablet houses the digital software that allows patients to record their vital signs, report on their experience while on the program, and communicate with the RCM management team. All devices are Bluetooth-enabled and are synched so that records of the patient’s vital signs are uploaded electronically to the software. Patients also have the option to manually upload data. At present, this software is licensed from a single provider, Aetonix™, which also provides storage and basic processing of all uploaded patient data.

All patient data are monitored and reviewed by members of the RCM team. Specifically, trends in patient data on vital signs and output from patient experience surveys are monitored. Recorded vital signs are reviewed daily. The digital software helps with this by identifying recorded measures that are outside of acceptable limits. This means that the RCM management team is alerted when a patient reports a blood pressure level, blood oxygen level, or weight that deviates from a desired range. If any or a combination of these events occurs, the clinical coordinator contacts the patient for a further assessment using the software’s call feature.

Standard parameters for patient vital signs are typically set at \[
\frac{{90\, - \,150}}{{60\, - \,100}}
\] mmHg for blood pressure, 60–100 bpm for heart rate, and 92–95% SpO2 for blood oxygen level. These parameters are consistent with medical practice within the Halton Region. The RCM management team is alerted by the software if these parameters are notably out of range. Specifically, alerts are triggered under any one of the following conditions:

1. A systolic blood pressure < 90 mmHg, or
2. A systolic blood pressure >150 mmHg, or
3. A diastolic blood pressure < 60 mmHg, or
4. A diastolic blood pressure > 100 mmHg, or
5. A heart rate < 60 bpm, or
6. A heart rate > 100 bpm, or
7. A blood oxygen level < 92% SpO2, or
8. Weight gain ≥ 2 lbs/day or weight loss ≥ 5 lbs/day from the patient’s baseline weight.

While the standard parameters for vital signs offer a benchmark for measuring patient conditions, these parameters can be adjusted to accommodate a patient’s baseline values and/or health goals. For example, some patients may generally have moderately lower or higher blood pressure levels at baseline that may not warrant medical attention. Likewise, cardiologists may recommend that some patients with CHF maintain a heart rate of 50–60 bpm with medication to avoid undue stress on the heart. Also, patients diagnosed with COPD typically have a lower target blood oxygen range of 88–92% SpO2 and measurements outside of this range will trigger an alert. Meanwhile, weight thresholds are modified for patients with CHF because of the nature of presentation of these respective diseases. In this regard, the software can be customized to accommodate the health needs of each patient so that alerts are not activated indiscriminately.

The assessment of a patient whose vital sign(s) prompted follow-up from the RCM management team will determine the next step in the flow of care. The following scenarios may arise:

1. The RCM management team may troubleshoot self-management issues with the patient in less severe events.
2. A representative from the community paramedicine program will be invited to conduct a wellness check with the patient.
3. The patient may be encouraged to visit their family doctor or cardiologist as a follow-up.
4. The patient may be encouraged to visit the emergency department.

The decision to recommend any one of these pathways is based on the RCM team’s clinical judgment of the patient’s current vital signs and physiological conditions.

Patient experiences while enrolled in the RCM program are captured using a questionnaire administered on a tablet at two intervals: after the first 7 days of the program and after the first 30 days of the program. Administering and reporting of survey results are requirements for Ontario Health. The survey questions are as follows:

1. My care **experience** was the same as or better than the care I would have received in person.
2. The program **empowers** me to be more active in **managing my health**.
3. I am **confident** that I will continue to manage my health at home.
4. The technology was **easy to use**.
5. I would **recommend** this program to a friend or family member.
6. Overall, how **satisfied** were you with the remote monitoring program?

Patients are encouraged to respond to each question based on the following Likert scale:

1. Very Dissatisfied
2. Dissatisfied
3. Neutral
4. Satisfied
5. Very Satisfied

Generally, communication between the RCM team and the patient (outside of onboarding, enrolment, and alerts/escalations) is as needed. Patients are encouraged to contact the RCM team with any concerns or questions they may have regarding the use of the devices and/or the remote care management software, or to discuss matters concerning self-care management or changes in symptoms. The RCM team is available Monday to Friday, 9:00 am to 5:00 pm. Outside of these hours, patients have access to a 24/7 hotline provided by Ontario Health atHome.

The program addresses several issues of equity and patient centricity. Firstly, the digital software patients interact with is accessible in seven languages: English, French, Spanish, Portuguese, Chinese (traditional and simplified), and Tamil. Communication with the RCM team is currently only in English; however, the intention is to provide translation services in the future. Secondly, during the onboarding process, patients are trained by the RRN on how to operate all devices, and translation services are provided as needed. Manuals and troubleshooting guides for each device are also shared with the patients. Thirdly, the tablet is equipped to accommodate both Wi-Fi and SIM connectivity. SIM connections are a necessary alternative, as some patients, including those in more rural areas, may not have access to the internet or even stable Wi-Fi connections. Fourthly, given that patients have more autonomy in managing their care, they also have unfettered access to records of their vital signs, which are uploaded to the software for future use. They can download and share these records with their family doctor, if needed. There is also no limit to the patient’s length of stay in the program. Patients are discharged when they feel sufficiently confident and comfortable to manage their condition without support from the RCM team.

### Involvement of those with lived experiences: patients, families, and caregivers

The program was designed with an emphasis on patient and caregiver engagement. Their involvement is reflected in key aspects, namely co-design, onboarding and training, flexible monitoring and communication, and empowerment through technology. Patients and their care partners, including family members and caregivers, were actively involved in the co-design of the RCM program. This participatory approach ensured that the program was tailored to meet the real-world needs and preferences of those receiving care. During onboarding, patients receive personalized training from RRNs on how to use the provided devices and software. Caregivers were also included in this process to support patients in managing their health at home. The program enables patients and caregivers to communicate with the RCM team via synchronous and asynchronous channels. This flexibility allows them to report symptoms, ask questions, and receive guidance as needed. Patients are equipped with Bluetooth-enabled devices and a tablet that facilitates automated data capture and communication. This setup enables patients and caregivers to monitor health indicators and engage with the care team proactively.

Lived experience was incorporated into the RCM program in several ways. Firstly, the patient-experience surveys serve as a feedback loop to help refine the program. Secondly, the digital software was made available in seven languages, and translation services were provided during onboarding. This ensures that patients from diverse backgrounds can participate and express their lived experiences without language barriers. Thirdly, the program emphasizes patient autonomy by allowing individuals to manage their care independently or with the support of a caregiver. Patients have full access to their health data and can share it with their physicians, reinforcing their role as active participants in their care journey.

## Evaluation of the CCHOHT chronic diseases RCM program

Table 3 provides a brief description of the features of the chronic diseases RCM program based on the RCM taxonomy characteristics. This is used as a reference for scores awarded in the evaluation process.

**Table 3:** Chronic diseases RCM program features based on the RCM taxonomy characteristics.


CHARACTERISTICS	OPERATIONAL DEFINITION

A1: Alert protocol	[Automatic] An alert is triggered when at least one recorded vital sign of a patient deviates from an established range.

A2: Data entry modality	[Fully automated] The tablet and all instruments used to measure vital signs are Bluetooth-enabled, and measurements are recorded electronically.

A3: Data access	[Centralized] Recorded measurements/collected data are managed by the software vendor and are accessible to the RCM monitoring team and patient.

A4: Manual data entry (frequency)	[Optional] Patients have the option to enter their vital signs measurements manually. This is not a requirement since the devices that they receive are Bluetooth-enabled, and measurements are recorded electronically. They also have the option to contact the RCM team via call or text using the software to report changes in symptoms if needed.

B1: Follow-up communication	[Synchronous & Asynchronous on demand] Patients have the option to contact the RCM team via call or text using the software to report changes in symptoms as needed. However, the RCM team is only available Monday to Friday between 9:00 am and 5:00 pm. Patients have the option to access other community resources outside of this time. Messages left for the RCM team outside of working hours are addressed on the next business day.

B2: Level of monitoring specialization	[Highly specialized] The core RCM monitoring team includes a physician, a rapid response nurse, nurses, paramedics, and respiratory therapists.

B3: Availability of RCM team	[Regular workdays] The RCM team is available Monday through Friday, 9:00 am to 5:00 pm. Patients have the option to access other community resources outside of this time.

B4: Risk profile	[Inclusive] There is no restriction on the severity of disease status for enrolment in the RCM program. However, the patient or their caregiver must be able to measure vital signs.

C1: Integration considerations	The RCM program is integrated with the following: Existing services and resources (i.e., shared full-time equivalent with existing services and programs) ☑ Existing workflows (e.g., embedded into usual clinical visits and procedures such as intake processes) ☑ Existing systems and infrastructure (e.g., patient records, EMRs, etc.) ☑

C2: Device linkages	[Single] The remote monitoring software is provided by a single vendor that is compatible with a single tablet manufacturer.

D1: Device ownership	[System provided] Patients are provided with all devices (including a tablet, pulse oximeter, blood pressure cuff, weight scale (for patients with CHF) and a SIM card if required) in a single package.

D2: Equity consideration	The RCM program promotes equity by: Promoting language inclusivity: The program is accessible in seven languages ☑ Promoting digital literacy (provides regular support to users with little education or digital literacy) ☑ Enabling offline functionality (does not require users to have constant internet access and/or allows data to be collected offline and synced at a later time) ☑ Adapting the program culturally (reports any considerations in making the RCM platform/program responsive to culture) ☒ Providing patients with digital access (provides all devices to the user and/or does not make the user possess a specific device or access to the internet as a pre-enrolment requirement) ☑ Enabling patients’ access to their own personal health information (interface allows patients to see their own data) ☑


The CCHOHT chronic diseases RCM program scored the same on both the OMH taxonomy rubric and the amended OMH taxonomy rubric. All but two program characteristics scored high using both tools. The program received a score of 2 for the following characteristics: B1- Follow-up communication and B2- Availability of team. The overall score was 2.875. See Table 4 in the main text and Table S4 in the supplementary file.

**Table 4:** CCHOHT chronic diseases RCM program scores using the OMH updated rubric.


DOMAINS/CHARACTERISTICS	HIGH (3)	MODERATE (2)	LOW (1/0)	TOTAL	AVERAGE

Domain A: Technology					

A1. Alert protocol	3			3	

A2. Data entry modality	3			3	

A3. Data access	3			3	

A4. Manual data entry (frequency)	3			3	

Domain B: Touch				12	3

B1. Follow-up communication		2		2	

B2. Level of monitoring specialization	3			3	

B3. Availability of team		2		2	

B4. Risk profile	3			3	

Domain C: Integration				10	2.5

C1. Integration consideration	3			3	

C2. Device linkages	3			3	

Domain D: Equity/Patient Centricity				6	3

D1. Device ownership	3			3	

D2. Equity consideration	3			3	

				6	2.5

				34	**2.875**


Based on the scores allocated for each program feature, the chronic diseases RCM program is described as having high technology, high touch, high integration, and high equity, occupying the uppermost left cell (cell 1A) of both the OMH RCM taxonomy matrix and the amended RCM taxonomy OMH matrix. This classifies the program as being Group A-Type 1. See Table 5 in the main text and Table S5 in the supplementary file.

**Table 5:** Updated OMH RCM taxonomy matrix according to type and group for CCHOHT chronic diseases RCM program.


			GROUP																	

		GROUP A			GROUP B		GROUP C		GROUP D		GROUP E		GROUP F		GROUP G		GROUP H		GROUP I	
								
			TECH	TOUCH	TECH	TOUCH	TECH	TOUCH	TECH	TOUCH	TECH	TOUCH	TECH	TOUCH	TECH	TOUCH	TECH	TOUCH	TECH	TOUCH

			High	High		High	High				High			High						

					Mod			Mod	Mod	Mod						Mod	Mod			

												Low	Low		Low			Low	Low	Low

TYPE	Type 1																			

	Integration	Equity	1A		1B		1C		1D		1E		1F		1G		1H		1I	

	High	High																		

	

	

	Type 2																			

	Integration	Equity	2A		2B		2C		2D		2E		2F		2G		2H		2I	

	High																			

		Mod																		

	

	Type 3																			

	Integration	Equity	3A		3B		3C		3D		3E		3F		3G		3H		3I	

		High																		

	Mod																			

	

	Type 4																			

	Integration	Equity	4A		4B		4C		4D		4E		4F		4G		4H		4I	

	

	Mod	Mod																		

	

	Type 5																			

	Integration	Equity	5A		5B		5C		5D		5E		5F		5G		5H		5I	

	High																			

	

		Low																		

	Type 6																			

	Integration	Equity	6A		6B		6C		6D		6E		6F		6G		6H		6I	

		High																		

	

	Low																			

	Type 7																			

	Integration	Equity	7A		7B		7C		7D		7E		7F		7G		7H		7I	

	

	Mod																			

		Low																		

	Type 8																			

	Integration	Equity	8A		8B		8C		8D		8E		8F		8G		8H		8I	

	

		Mod																		

	Low																			

	Type 9																			

	Integration	Equity	9A		9B		9C		9D		9E		9F		9G		9H		9I	

	

	

	Low	Low																		


## Discussion

The CCHOHT chronic diseases RCM program is shaped by engagements and collaborations with patients, clinicians, and caregivers, which ultimately influence the administration of remote management. The program is based on the CCHOHT’s model of remote management. It was developed as a digital intervention that remotely manages patients, provides follow-up communication, and escalates critical cases through a more integrated clinical pathway of services and care partners for patients with selected chronic conditions, including COVID-19, COPD, CHF, pneumonia, ILD, asthma, and pulmonary embolism.

The program also meets the Ontario Health RCM taxonomy criteria of technology touch, integration, and equity, attaining a maximum score of 3 for all but two program characteristics. A score of 2 was awarded to the following characteristics: B1- Follow-up communication and B2- Availability of team. We provided a detailed description of the program as evidence to support the scores awarded.

We proffer the following justification for allocating less than the maximum score for the two characteristics mentioned above. Regarding touch, there are protocols for communication between the RCM team and patients during patient onboarding and for medical escalations. Patients are encouraged to contact the RCM team as needed. Still, there is no expectation that the team will contact patients periodically for general wellness and management checks. Additionally, the RCM team is only available Monday to Friday from 9:00 am to 5:00 pm (except on holidays). Patients requiring intervention outside of these times are encouraged to access alternative resources. It is anticipated that by addressing the issues surrounding the team’s availability and scheduled check-ins with patients, the program’s score on touch can likely improve.

While the program is implicitly rooted in WHO’s principle of the right to health around non-discrimination, physical accessibility, economic accessibility (affordability), and information accessibility [12], there is scope to affirm cultural sensitivity approaches to care within the RCM team, given the diverse population in Halton Region. This population includes large groups of people of Southeast Asian descent, Indigenous groups, and a growing migrant population [13].

The evaluation performed in this study demonstrates how RCM programs can be developed by accounting for key components that can potentially lead to overall program success. It also contributes to the growing evidence on guidelines for building RCM programs, especially in Canada, and so presenting the CCHOHT’s RCM model and chronic diseases RCM program provides a template that other OHTs and health organizations can follow, albeit with accommodations for context-specific circumstances.

The program’s features also align with the six remote monitoring success factors outlined by Thomas et al. (2021) in their realist review, namely, targeting populations at high risk, accurately detecting a decline in health, providing responsive and timely care, personalizing care, enhancing self-management, and ensuring collaborative and coordinated care [2]. Program enabling factors, as prescribed by CADTH, specifically, patient engagement, patient experiences, and a team-based approach to care [1], are also built into the chronic diseases RCM program. These features are also aligned with some of the pillars of integrated care, namely, shared values and vision, population health and local context, people as partners in care, workforce capacity and capability, new alliances, digital solutions, and transparency of progress, results and impact [14].

It is anticipated that the program will achieve the benefits materialized by other documented RCM programs, namely, optimal patient care and effective treatment [15]. One quantifiable benefit is a reduction in the utilization of in-person clinical services, including ED visits, physician visits, and hospital admissions [4,5,16]. This ultimately has implications for minimizing health costs incurred by patients and the broader health system [5]. Improvements in patient, family, caregiver, and clinical worker experiences in the delivery of care are also anticipated [4]. Remote management also provides an opportunity for individuals who face barriers to accessing in-person care, be it from distance or socioeconomic factors, to have a direct channel of communication with a clinician and/or care team, ensuring equity issues are addressed [17,18]. It was noted to be a safe and feasible outpatient care strategy, with endorsements from both patients and clinicians that programs were both comforting and beneficial [7,17,19].

The CCHOHT RCM program architects are keen on avoiding pitfalls that may impede the program’s success. As prescribed by Kirkland et al. (2023), particular attention will be given to addressing concerns related to patients, providers, the health system, the digital infrastructure, and intervention design [17]. An integral part of the process calls for continuous focus on resourcing needs (financial and staffing), leadership, improving communication and coordination among program participants, providing the necessary supports (for patients, caregivers, families, and the clinical team), and ensuring reliable information technology systems [17,19].

Implementing an RCM program also requires consideration of standards, namely clinical effectiveness and cost effectiveness, through which RCM tools can be evaluated before they are integrated into care pathways [5]. This can only be proven through appropriate research endeavours, such as randomized controlled trials, prospective observational studies, and economic evaluation studies. Such studies would also help identify populations that would be most suitable for RCM interventions, where the full benefits of such a program can be realized [5]. Another concern is that remote management may not be ideal for more debilitated patients, as in-person visits with a physician may be preferred for such persons [2]. At the same time, remote management is expected to complement rather than replace in-person physician visits [20]. In this regard, how RCM can be adapted to accommodate more critical patients should also be considered, as program benefits are more pronounced for cohorts with a longer life expectancy [2].

As more RCM programs are developed across the province and the country, we encourage program organizers to engage in similar evaluation exercises. We reemphasize that evaluating the structure of an RCM program assesses whether the foundations of integrated care are rooted in the program. We anticipate that remote management would complement rather than completely replace other forms of in-person care, especially since there is a growing demand for this service among Canadians [20].

This study has some limitations. The main limitations surround the evaluation metric forwarded by the OMH. For example, parameters for some moderate categories were not defined in the rubric, which makes the rubric unbalanced and introduces bias in scoring. Furthermore, the taxonomy does not allow for a moderate score, although this option is provided in some cases in the rubric. Also, no benchmark characteristics were provided to define intervals for high, moderate, and low scores in the overall evaluation. The amended taxonomy and rubric proposed in this study sought to address these challenges, although resulting in an expanded taxonomy matrix with notably more descriptive categories. These additional categories were rationalized to make the matrix more user-friendly. The developers of the original taxonomy also acknowledged that touch, technology, integration, and equity are by no means an exhaustive list of RCM evaluation criteria and that other criteria, such as sustainability and scalability, can also be considered [4]. Another notable limitation is the likely inherent bias of self-evaluation. The program coordinators acknowledge this challenge but affirm that the description of the program provided in this manuscript will allow reviewers to conduct independent evaluations. Additionally, we acknowledge the potential bias inherent in using only an intuitive approach to rescale the rubric. Although this decision was feasible, future iterations of the RCM tool can consider incorporating more rigorous quantitative and qualitative methods to develop the rubric.

As a next step, we intend to share the updated taxonomy and rubric with the OMH. We hope that the ministry will support the provincial-wide use of these tools among other agencies with RCM programs. The ministry’s support is also welcomed for future improvements of the tools. We anticipate that with the provincial government championing the tools, other stakeholders, particularly those with lived experiences (namely, patients, families, and caregivers), would feel confident in participating in future developments of the tools and evaluation exercises. In the meantime, the CCHOHT is pursuing other forms of evaluation for its chronic diseases RCM program, including patient-reported outcomes and experiences, and health system impact (i.e., clinical effectiveness and cost-effectiveness analyses). The organization is also exploring ways to expand the RCM program to other priority conditions in the region, such as diabetes, palliative care, and wound care.

### Recommendations

We propose several recommendations to enhance the program’s impact, scalability, and alignment with integrated care principles based on the literature and our experience with RCM. We proffer that an RCM program should be developed with the following core features: a fully integrated digital platform with connectivity that facilitates remote monitoring; coordinated services along each care pathway to improve the experiences of patients and families/caregivers; greater involvement of these stakeholders in the design and execution of care management strategies, ultimately promoting greater patient autonomy; seamless connectivity between patients, families/caregivers and the remote monitoring team, and built-in mechanisms to ensure greater equity and access to the program.

On a granular level, we propose the following additional recommendations. To integrate lived experiences, we recommend establishing structured feedback loops with patients and caregivers throughout the design, implementation, and evaluation phases. This includes incorporating testimonials or case studies that reflect lived experiences to enrich program insights and engaging patient advisory panels to guide program enhancements and ensure cultural responsiveness. To improve patient engagement, enhanced availability of the care team, and care continuity, we propose the introduction of scheduled wellness check-ins by the RCM team, expanded communication hours to ensure responsiveness beyond standard business hours either through direct staffing or partnerships with community paramedicine programs, and automated reminders and alerts to prompt patient engagement and symptom reporting. To improve equity standards, the following strategies can be considered: offering multilingual support throughout all phases of care, ensuring cultural safety training for all RCM team members, and developing tailored onboarding materials for diverse literacy levels and accessibility needs. We also proposed a fifth principle called outcomes to measure the main achievements of RCM programs.

## Conclusion

We described the CCHOHT’s new chronic diseases RCM program and demonstrated how it aligns with the Ontario Ministry of Health’s principles for remote care and integrated service delivery. The program is described as being fully automated and integrated with existing care pathways. It promotes greater patient inclusion and autonomy in home care, supported by continuous monitoring and frequent interactions between patients and the RCM teams.

Based on the review of the program, its high scores across technology, integration, and equity domains affirm its structural soundness and potential for replication. Importantly, the program’s adaptability is not merely theoretical—it is evidenced by its current application to chronic disease management and its planned extension to palliative and wound care pathways. This flexibility positions the model as a scalable solution for diverse clinical contexts, particularly in regions seeking to enhance patient autonomy, reduce hospital admissions, and improve care coordination.

The amended evaluation tool proposed in this study addresses key limitations in the original taxonomy by introducing balanced scoring, clearer descriptors, and a more nuanced classification matrix. This enhanced framework provides a practical and replicable method for developing RCM programs, serving as a valuable resource for other Ontario Health Teams and health organizations worldwide.

While the program has demonstrated strong alignment with integrated care principles, opportunities remain to strengthen its responsiveness and inclusivity. Specifically, formalizing the integration of lived experience and expanding team availability will further enhance its impact. Nonetheless, the CCHOHT RCM model offers a compelling example for remote care delivery that is patient-centred, technologically advanced, and grounded in equity.

## Additional File

The additional file for this article can be found as follows:

10.5334/ijic.8629.s1 Supplementary File. Tables S1–S5.
