# Supplementary material for: An Evaluation of the Structure of an Integrated Regional Remote Care Management Program for Patients with Selected Chronic Diseases in Canada
Source: Int J Integr Care. 2026 Feb 5;26(1):4. doi: 10.5334/ijic.8629 (PMC12880051; doi:10.5334/ijic.8629)
Supplement: Supplementary File. — Tables S1–S5. [file ijic-26-1-8629-s1.pdf]

An evaluation of the structure of an integrated regional remote care management program for patients with selected chronic diseases in Canada

Diedron Lewis[1], Karin Swift[2], Sarah Weberman[2]

[1] University of Toronto, Ontario, Canada
[2] Connected Care Halton Ontario Health Team, Halton, Ontario, Canada

Supplementary Material

Method

The Ontario Ministry of Health RCM taxonomy was developed by observing patterns in existing RCM programs identified in a review of 87 articles selected from two environmental scans of the literature. The first literature search focused on chronic disease RCM programs, namely for hypertension, diabetes, chronic obstructive disease, and congestive heart failure, while the second search focused on RCM programs for COVID-19. Consultations with clinicians and other persons with relevant experiences in remote care management also supported the literature scan and review. See "Remote Patient Monitoring: Evaluation Final Report" for the methodology on constructing the RCM taxonomy [4].

The RCM taxonomy comprises 12 characteristics, grouped into four domains: A = Technology, B = Touch, C = Integration, and D = Equity/Patient-Centricity [4]. Table S1 describes these characteristics and domains.

Table S1. RCM glossary of taxonomy characteristics

**Domain**	**Characteristics**	**Operational Definition**
A: Technology<br><br>Refers to the level of automation and technical complexity of the RCM platform.	A1: Alert protocol	Refers to how a decision is made to conclude that escalation is needed. Escalation can mean that the RCM/clinical team contacts the patient or the patient calls 911 or reaches out to other supportive services.
	A2: Data entry modality	Describes how patient physiologic measurements or other assessments are collated and/or reported. This excludes symptoms, PREMs/PROMs, and patient experience surveys, which are always entered manually.
	A3: Data access	Describes how measurements/collected data from the RCM program is streamlined or managed from the point of entry, where it is reviewed, or decisions are made.
	A4: Manual data entry (frequency)	Refers to the frequency for which a patient is required to manually enter data (i.e., measurements that are not automatically captured, symptoms, PROMs/PREMs, experience surveys). This excludes measurements that are

		automatically captured.
B: Touch<br><br>Refers to the level of monitoring and interaction required between the patient and the RCM team.	B1: Follow-up communication	Describes how the RCM team stays connected or follows up with the user. Refers to general two-way communication between the clinical team and the patient. This excludes communication regarding onboarding, enrolment, and alerts/escalations.
	B2: Level of monitoring specialization	Describes the level of clinical specializations of the RCM monitoring team. The RCM monitoring team refers to individuals who regularly monitor patient data, communicate with patients, and/or are available to respond to clinical escalations.
	B3: Availability of RCM team	Describes the availability of the RCM monitoring team to respond to patient inquiries, alerts, and clinical escalations.
	B4: Risk profile	Refers to the severity of disease status for people enrolled into the RCM program.
C: Integration<br><br>Refers to the extent to which the RCM program is linked to (or leverages) existing systems (i.e., interoperability) including services, resources, workflows, and infrastructure.	C1: Integration considerations	Considers whether the RCM program is integrated with the following:<br>1) existing services and resources (i.e., shared full-time equivalent with existing services and programs),<br>2) existing workflows (e.g., embedded into usual clinical visits and procedures such as intake processes), and<br>3) existing systems and infrastructure (e.g., patient records, EMRs, etc.)
	C2: Device linkages	Considers the linkages between individual hardware and software devices used in the RCM program (e.g., platform vendors, cloud

		services, digital applications, etc.) irrespective of device manufacturer or vendor. For example, a multiple integrated program will have >1 device serviced by >1 manufacturer/vendor, but still be designed to function as a single unit.
D: Equity/Patient Centricity<br><br>Refers to the extent to which the RCM program proactively enables inclusion, equitable access, and/or patient-centricity.	D1: Device ownership	Refers to the extent or burden on the patient with respect to device ownership. This includes any device required to participate in the program, such as physiologic devices, non-physiologic devices for alerts/escalations, a phone, tablet, laptop, etc.
	D2: Equity consideration	Considers whether the RCM program promotes equity by:<br>1) promoting language inclusivity (provides program in >1 language),<br>2) promoting digital literacy (provides regular support to users with little education or digital literacy),<br>3) enabling offline functionality (does not require users to have constant internet access and/or allows data to be collected offline and synced at a later time),<br>4) adapting the program culturally (reports any considerations in making the RCM platform/program responsive to culture),<br>5) providing patients digital access (provides all devices to the user and/or does not make the user possess a specific device or access to the internet as a pre-enrolment requirement), and<br>6) enabling patients access to their own personal health information (interface allows patients to see their own data).

Source: Centre for Digital Health Evaluation, Women's College Hospital Institute for Health System Solutions and Virtual Care (2023) "Remote Patient Monitoring: Evaluation Final Report"

Table S2. RCM taxonomy matrix according to type and group resulting in 16 program typologies

			GROUP							
			Group A		Group B		Group C		Group D	
			Tech	Touch	Tech	Touch	Tech	Touch	Tech	Touch
			High	High		High	High			
					Low			Low	Low	Low
TYPE	Type 1									
	Integration	Equity	1A		1B		1C		1D	
	High	High								

	Type 2					
	Integration	Equity	2A	2B	2C	2D
	High					
		Low				
	Type 3					
	Integration	Equity	3A	3B	3C	3D
		High				
	Low					
	Type 4					
	Integration	Equity	4A	4B	4C	4D
	Low	Low				

Source: Centre for Digital Health Evaluation, Women's College Hospital Institute for Health System Solutions and Virtual Care (2023) "Remote Patient Monitoring: Evaluation Final Report"

Table S3. Rubric for evaluating RCM domains and characteristics

Domains/Characteristics	High (3)	Moderate (2)	Low (1/0)
Domain A: Technology			
A1. Alert protocol	[Automatic]	N/A	[Manual] or [None] or [Unknown]
A2. Data entry modality	[Fully automated] or [None]	[Semi-automated]	[Manual] or [Unknown]
A3. Data access	[Centralized]	N/A	[Fragmented] or [None] or [Unknown]
A4. Manual data entry	[Monthly] or [None]	[Weekly] or [Bi-	[Daily] or [Unknown]

(frequency)		weekly]	
Domain B: Touch			
B1. Follow-up communication	[Synchronous on demand]	[Asynchronous on demand]	[Pre-scheduled] or [None] or [Unknown]
B2. Level of monitoring specialization	[Moderately specialized]	N/A	[No specialization] or [None] or [Not applicable
B3. Availability of team	[24/7] or [Regular + weekends]	[Regular workdays]	[Irregular] or [None] or [Not applicable] or [Unknown
B4. Risk profile	[High]	[Moderate]	[Low] or [Non-specific] or [Unknown]
Domain C: Integration			
C1. Integration consideration	[3]	[2]	[1] or [None] or [Unknown]
C2. Device linkages	[Multiple linked] or [Single]	N/A	[Multiple separate] or [None] or [Unknown]
Domain D: Equity/Patient-Centricity			
D1. Device ownership	[System provided]	[Mixed ownership]	[BYOD] or [None] or [Unknown]
D2. Equity consideration	[5] or [6]	[3] or [4]	[1] or [2] or [None] or [Unknown]

Source: Centre for Digital Health Evaluation, Women's College Hospital Institute for Health System Solutions and Virtual Care (2023) "Remote Patient Monitoring: Evaluation Final Report"

Table S4. CCHOHT chronic diseases RCM program scores using the OMH rubric

Domains/Characteristics	High (3)	Moderate (2)	Low (1/0)	Total	Average
Domain A: Technology					
A1. Alert protocol	3			3	
A2. Data entry modality	3			3	
A3. Data access	3			3	

A4. Manual data entry (frequency)	3			3	
				12	3
B1. Follow-up communication		2		2	
B2. Level of monitoring specialization	3			3	
B3. Availability of team		2		2	
B4. Risk profile	3			3	
				10	2.5
C1. Integration consideration	3			3	
C2. Device linkages	3			3	
				6	3
D1. Device ownership	3			3	
D2. Equity consideration	3			3	
				6	3
				34	**2.875**

Table S5. OMH RCM taxonomy matrix according to type and group for CCHOHT chronic diseases RCM program

			GROUP							
			Group A		Group B		Group C		Group D	
			Tech	Touch	Tech	Touch	Tech	Touch	Tech	Touch
			High	High		High	High			
					Low			Low	Low	Low
TYPE	Type 1									
	Integration	Equity	1A		1B		1C		1D	
	High	High								
	Type 2									
	Integration	Equity	2A		2B		2C		2D	

	High					
		Low				
	Type 3					
	Integration	Equity	3A	3B	3C	3D
		High				
	Low					
	Type 4					
	Integration	Equity	4A	4B	4C	4D
	Low	Low				
